# Learning-Based Ordering Characters on Ancient Document

**DOI:** 10.1155/2022/3260384

**Published:** 2022-11-17

**Authors:** Hyeonjin Lee, Rock-Hyun Baek, Hyun-Chul Choi

**Affiliations:** ^1^ICVS Lab, Department of Electronic Engineering, Yeungnam University, 280 Daehak-Ro, Gyeongsan, Gyeongbuk 38541, Republic of Korea; ^2^Department of Electrical Engineering, Pohang University of Science and Technology, Pohang, Gyeongbuk 37673, Republic of Korea

## Abstract

Digitalizing and translating a scanned document image entails detecting the characters using a detector and translating the characters in the order they were detected with a translator. However, it is impossible to translate these characters correctly because the detector often detects them in any order. As a result, since it is critical to organize the recognized characters for proper translation, we propose ordering characters from documents with multiple variations using the strength of the learning-based model that learns the necessary operations from the data. In this task, it is difficult to order the characters written on antique handwritten documents that have deviations such as a bent or split line, as opposed to official records that have lines placed uprightly one by one. Because dealing with these many variants using a human-designed algorithm is problematic, we arrange characters printed on papers with diverse variations by taking advantage of a training model that can learn the appropriate function from data. Our method outputs both line id and *y*-axis and combines them to assign the sequential index. It is difficult to train using simply local regions because sequential character indexes in a large range include long-range dependencies. To solve this problem, we use network architecture to expand the receptive field as wide as possible. The network must learn to give various indexes to characters in similar places for each document because the number and area of characters vary for each document. We offer the ground truth assign method based on the absolute position to assign similar indexes to characters in similar places. Furthermore, even if the network uses absolute ground truth, the network may assign the incorrect line if the center coordinates of characters are biased in one direction. As a result, we employed the Region of Interest (ROI) from the pretrained coordinate layer, which contains position and trend information. We used the modified edit distance to compare the similarity of character indexes from the ground truth and our technique. In addition, we computed the modified fisher criterion to assess the degree of the clustering line. Consequently, our edit distance is just 0.43 times that of the human-designed algorithm, and our fisher criterion is 1.46 times that of the human-designed algorithm, improving the performance of human-designed algorithm.

## 1. Introduction

Digitalization is the process of converting characters on scanned documents or photographs into digital data, which has made it possible to search for document content, perform statistical analysis, create high-quality content, send or receive information, and support the creation of digital archives. Optical character recognition (OCR), which automatically enters all characters in photographs into a computer without the typing of human, is a typical example of digitalization. We computerize vast amounts of image data quickly and efficiently by utilizing this feature, saving significant time and storage space in managing large-capacity paper documents. Digitalization is also useful for translating foreign papers. The processing procedure is to first output the coordinate values of the detected character region and the probability of the presence of the characters and then to input the detected characters into the translator to perform translation. However, the characters are input to the translator in an order that is unrelated to the translation because there is no order value for each detected character that corresponds to the translation, and this may result in incorrect translation.

To the best of our knowledge, there is no existing work that assigns a corresponding order to text translations. Of course, there are some works dealing with document layout, such as text line extraction [1, 2], text line segmentation [3, 4], and scene text detection [5]. There are also several works on recognizing characters such as Gurmukhi [6–11] and Devanagari [12], as well as a work on machine translation [13].

**Figure 1:**
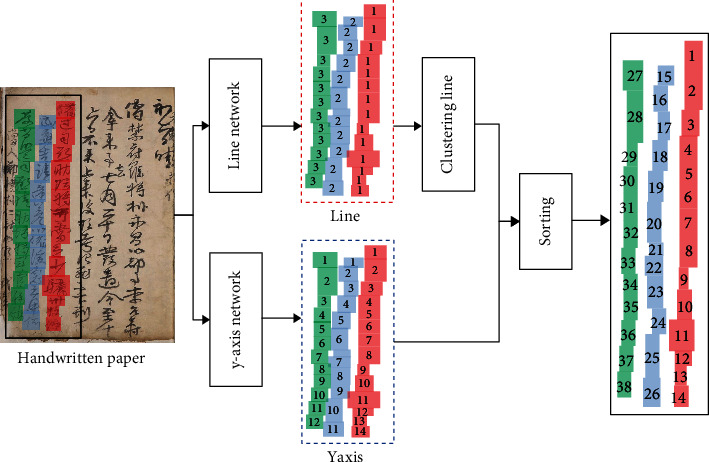
The character ordering process of 2-dimensional indices.

We need to assign orders to the detected characters for correct translation because putting them into the translator in random order causes translation problem. Since there is no related prior research, we define a character reordering approach based on a learning network that detects patterns in the data on its own.  Figure 1 is a schematic diagram of the character ordering process. We assign orders separated in line and *y*-axis after putting an old document picture data to the network and then assign a series of orders using line clustering and a rearrangement procedure. In addition, we calculate the edit distance and the fisher criterion to assess the accuracy of the order, and we show that our system outperformed the nonlearning-based method on all measures.

Our contributions are as follows in brief:

- We define a new problem by dealing with tasks that have not been preceded so far and propose the method for ordering characters in a document using a learning model that learns necessary operations from data.
- The network infers the order value into two dimensional indices, line and *y*-axis, to increase the amount of learning information for correct learning.
- We propose a ground truth (GT) generation approach in which characters in similar positions have a similar order independent of the document and convey the order trend by injecting the coordinates and size of the character region into the index layer to make network learning easier.
- Ours outperformed the human-designed algorithm in the edit distance and fisher criterion accuracy. In particular, ours surpassed on split lines because our learning-based network has high clustering performance.

## 2. Background

We can presume that our working, character ordering, is a spatial relation learning since it assigns line ids by inferring left-right relations of characters and assigns *y*-axis ids in the same line by inferring the top-down relation. Relation learning has previously been widely explored as visual relation learning which deals with interactions such as inferring visual object-object [14] or human-object relations [15, 16]. This work relates to image understanding tasks that mix computer vision and natural language such as image retrieval [17], image captioning [18–20], and visual question answering [21]. It also infers relationships such as relative position (“behind, above”), action (“eat, ride”), and comparison (“taller than”) [22, 23].

Relationship inference has been carried out as a task with each visual relationship represented as a triplet in the form of subject-predicate-object [24–27]. For example, Sadeghi and Ali[25] proposed training a complex visual composite such as person-riding-horse to improve the localization of person and horse. These visual phrases make it easier to detect items since they consider the appearance changes and occlusions caused by different views and the interaction between objects. Learning the triplets as independent classes, on the other hand, has the limitation that it cannot be conducted on a big data set since the combinations of items are too diverse.

Recent works offered a strategy for learning objects and predicates separately and combining them to infer relational inferences even on huge data sets. If two or more tuple sets (e.g., truck-on-street and car-on-street) share the same predicate, they are classified as belonging to the same category [28]. However, combining one predicate with multiple objects increases the variability and can result in an issue that does not train well [29]. As a result, new research has presented a model for inferring relationships with statistical dependency using graphical models [30, 31], language distillation [32], or semantic context [33].

Zhang et al. [34] presented a strategy for dealing with combinatorial complexity by mapping subject and object into a low-dimensional relation space and modelling a relation triplet as a relation translation vector, i.e., subject + predicate ≈ object. For example, the network creates a consistent relational translation vector independent of subject (e.g., person) or object (e.g., horse, bike) given the riding photos. This method allows relational inference by learning only the relation translation vector in relation space rather than learning the many appearances of the triplet, even if the subject and object are very diverse.

When the attributes of each object are clearly defined, these methods can be useful. However, it is difficult to assign the correct ids in the same way as other approaches because the properties of objects are the same as characters in the ancient document in our character ordering task.

Some research studies [35, 36], on the other hand, define the relationship between symbols by combining language logic and mathematics to infer the relationship. Relation Networks (RNs) [37] proposed inferring the relationship between various pixels because creating associations between symbols is difficult. In this case, RN infers a potential relationship by examining the relationship between all pixel pairings regardless of whether an item exists in one pixel or whether there is a significant association.

Without knowing the properties of the object, this algorithm can deduce the relationship by evaluating all pixel combinations. However, the number of permutations between pixels grows rapidly when the feature map size grows, resulting in limitations of high memory utilization and waste due to meaningless pixel combinations. Also, there are some methods such as text line extraction [1, 2], text line segmentation [3, 4], scene text detection [5], machine translation [13], and recognizing characters such as Gurmukhi [6–11] and Devanagari [12]. But they do not propose the assigning translation order and do not evaluate the ordering performance like our method does. So, comparing ours to these related works is not appropriate.

## 3. Method

Character ordering is the problem of inferring the spatial relationship of characters within a document. The ancient documents we deal with are written line by line from left to right and top to bottom. As a result, this section describes how to identify the character order that corresponds to the translation order in the document by considering the left-right (line) and top-bottom (*y*-axis) order of characters.

### 3.1. Two Dimensional Indices and Network

**Figure 2:**
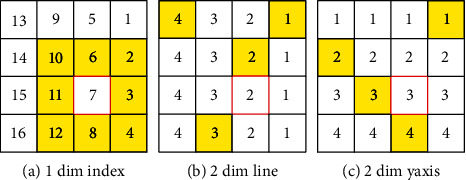
Effectiveness of 2-dimensional indices: The amount of information available for learning varies depending on the dimension of the index. (a) uses one combination (yellow boxes) to learn the order value (red box) but (b and c) use *S*^*S*^ combinations as information.

We propose a method to increase the quantity of information during the learning process which learns 2-dimensional indices (2-dim indices) separated into line and *y*-axis as illustrated in Figure 1 rather than 1-dimensional indices (1-dim indices).  Figure 2 depicts the variation in information amount based on the number of dimensions. We can only utilize one combination (yellow box) for learning one index (red box) for 1-dimensional indices (Figure 2(a)) which requires examining all nearby characters. 2-dimensional indices (Figures 2(b) and 2(c)), on the other hand, can employ *S*_*h*_^*S*_*w*_^(*S*_*w*_^*S*_*h*_^) combinations (yellow box) to learn one line or *y*-axis (red box) by picking data line by line or *y*-axis by *y*-axis. *S*_*w*_ and *S*_*h*_ represent the width and height of the feature map, respectively. When we partition the learning attribute in this way, we reduce the learning range while increasing the amount of learning information. As a result, the network may learn the order values.

**Figure 3:**
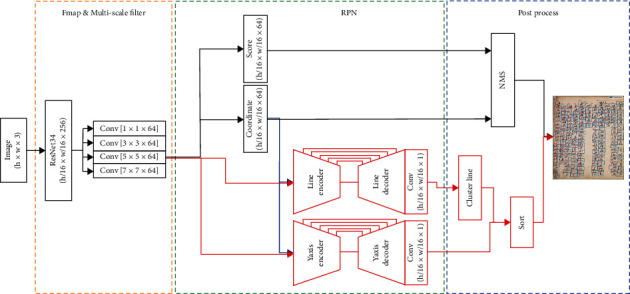
Network architecture and postprocessing for character ordering. Our network consists of Fmap and multiscale filter (yellow box) and RPN (green box). The index outputs from the 2-dimensional layer of RPN are rearranged in a series order through postprocessing (blue box).

For 2-dim indices character ordering, we added two index layers to RPN network-based faster R-CNN object detection [38] as illustrated in Figure 3. These index layers generate 2-dimensional indices. The first element (line encoder and decoder) represents the lines which is the ascending value from right to left in the whole document image according to the translation order illustrated in the Figure 1 red dash box. The second element (*y*-axis encoder and decoder) represents the order which is increasing from top to bottom inside a single line as indicated in the Figure 1 blue dash box.

Learning with a small receptive field of the convolution kernel is challenging because these two values are organic and consecutive throughout the document. For example, if an ancient text has ten lines, the restricted kernel size, 3 × 3, learns the line values 3, 4, and 5 and then slides to learn the line values 4, 5, and 6. However, the final line value to be assigned is 1 to 10. In other words, it is difficult to predict order values with long-range dependencies because the kernel accommodates a limited area of feature maps. As a result, we stack the pooling layer to increase the receptive field until the size of the feature map is smaller than the size of the kernel. The feature map is too small to match the GT shape and calculate the loss after downsampling to accommodate the full document in this manner. To construct the final feature map for use in the loss computation, we expand the size of the feature map by upsampling and reinforce the lost information by utilizing skip connection [39].

The character ordering, we will do necessitates understanding spatial relationships, but the corresponding approaches [30, 37] have drawbacks such as requiring object properties or a large amount of memory. So, we stack pooling layers to broaden the receptive field and infer spatial relationships such as left-right and top-bottom in a memory-efficient manner without distinguishing the attributes of objects rather than using combinations of objects or pixels to infer spatial relationships such as left-right and top-bottom.

### 3.2. Absolute Ground Truth

**Figure 4:**
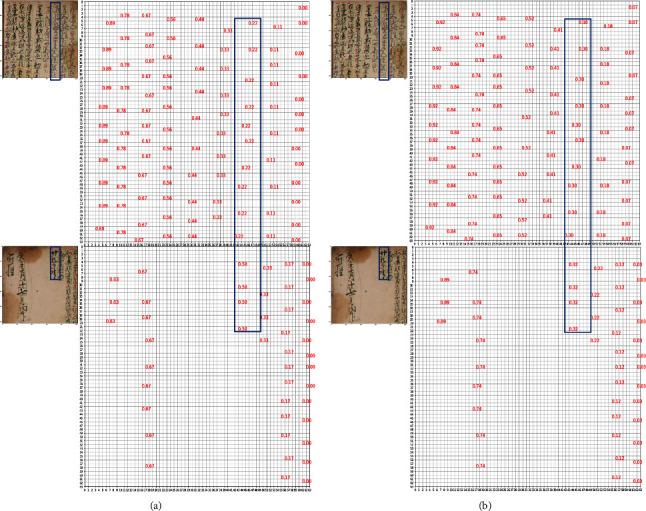
Original ground truth (left) and absolute ground truth (right). (a) Original ground truth. (b) Absolute ground truth.

We should define a loss function between the prediction and the ground truth (GT) to optimize the network. Assigning the ground truth value just to the spot where the character exists as with the GT map used for ROI learning [40–42], causes difficulty labeling different values at similar locations (Figures 4(a) and 4(b) in sec. Effectiveness of absolute ground truth) because various documents contain variable amounts of characters and areas filled by characters.

**Figure 5:**
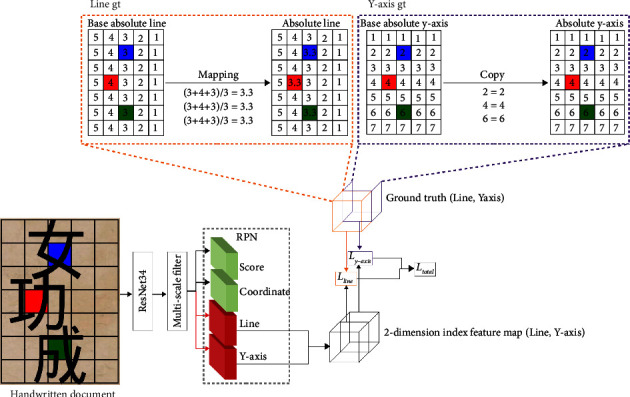
Absolute ground truth generation method: generates a base absolute line and a base absolute *y*-axis with consecutive numbers assigned to all pixel locations, even in the background where no characters exist. The white boxes of the line GT and the *y*-axis GT represent the background, while the blue, red, and green boxes represent each character of the handwritten document. Following the selection of the location in the generated mask where the character exists, we used the average of the selected values for each line as the absolute line and the selected values precisely as they are as the absolute *y*-axis.

As a result, we propose an absolute GT approach that assigns consecutive numbers to all pixel regions, including the background area where there are no characters. And we only use the locations where characters exist as GT.  Figure 5 shows an example of generating GT using the way we proposed. The white boxes in the line GT and *y*-axis GT represent the background in this case, while the blue, red, and green boxes represent each character in the handwritten document. The goal of this GT is to generate a 2-dim absolute GT map for three consecutive characters (blue, red, and green boxes) in the image of an ancient handwritten document (input image in Figure 5). The original line in the orange dashed box of Figure 5 is created by the procedure used to generate the existing ROI GT [40–42]. However, there is an issue with characters in similar positions in the two different input images having different line id GT values. To solve this, first, draw a base absolute line that allocates consecutive numbers in the same order as the translation order from right to left for each column. Following that, the absolute line is determined by taking the average of the values chosen for each line because the line ids may not be placed on a straight line on the feature map depending on the center coordinates of the character. This absolute line is assigned a similar line GT at a similar location regardless of the document. The absolute *y*-axis (purple dashed box in Figure 5), which is generated in the same way as the absolute line, has a sequential order from top to bottom for each column, and we do this by taking the translation order into account. Similar values can thus be labeled at similar locations allowing networks to learn similar values at similar locations.

### 3.3. ROI Transfer

If the center coordinates of some characters in a specific document are skewed in another direction, the network may infer a value that differs from other values in the same line even using Absolute GT. To address this issue, we offer an ROI transfer approach that adds the coordinate and size of the objects (*x*, *y*, width, and height) extracted from the ROI network to the line and *y*-axis encoders (blue lines in Figure 3). The coordinate information of the inserted ROI gives a tendency to assist in assigning ids in the translation order. If these variables have similar values, the network assigns similar ids, which aids in line clustering.

The feature map extracted from the feature extractor is now referred to as a graphical input feature map (GIF) because it contains graphic information. The one with the detection box information extracted from the coordinate layer is referred to as an ROI input feature map (RIF), and the one that concatenates these two feature maps is referred to as a GIF + RIF.

### 3.4. Objective Loss and Post Processing of Line Clustering

We compute the line and *y*-axis losses (*L*_line_ and *L*_*y*−axis_) from the network output to train the index layer. Equations (1) and (2) are L2 distances calculated by dividing the difference between the network output order (line, *y* − axis) and GT (line_*t*_, *y* − axis_*t*_) by the number of characters in the document (N). And equation (3) is a weighted sum of these two losses using *λ*_line_ and *λ*_*y*−axis_.

(1) $$\begin{array}{c} L_{line}=\frac{{\left(line-{line}_{t}\right)}^{2}}{N}\text{,} \end{array}$$

(2) $$\begin{array}{c} L_{y-axis}=\frac{{\left(y-axis-y-{axis}_{t}\right)}^{2}}{N}\text{,} \end{array}$$

(3) $$\begin{array}{c} L_{inde\;x}=\lambda_{line}\cdot L_{line}+\lambda_{y-axis}\cdot L_{y-axis}\text{.} \end{array}$$

After training the network to minimize index loss (*L*_index_), we cluster the lines that satisfy equation (4) where threshold is the experimentally established hyper-parameter and then rearrange them in translation order so that the line and *y*-axis, the network output, have consecutive integer values. Then, we rearrange the order for each line using the *y*-axis value predicted by the network, and we generate a series of integer values.

(4) $$\begin{array}{c} \;|\;{line}_{i:N-1}-{line}_{i+N:1}\;|\;<threshol\;d,i=1.N-1\text{.} \end{array}$$

## 4. Experiments

This section examined the effects and physical meanings of our learning-based character ordering network by gradually adding the components indicated in the method. We investigated the reasons for using 2-dimensional (2-dim) indices, the effect of absolute ground truth, and the effect in conveying ROI information. We defined the human-designed algorithm because there was no previous study to compare it to. To evaluate the performance of the two methods, we compared the edit distance (ED) [43] and Fisher criterion (FC) [44] values.

### 4.1. Experimental Setup

We used the coordinate and score layers of a multiscaled faster R-CNN [38] (Figure 3 yellow and green box) to detect all characters in the ancient document independent of character size.

We obtained 150 handwritten documents from the Korean Studies Institute [45] and used 40 for training, 10 for validation, and 100 for performance evaluation as a dataset. And the number of words in each paper varies between 10 and 450 for each document. The characters in these ancient documents are written vertically and line by line. Each line is ordered in ascending order from right to left, and the *y*-axis values in each line are arranged in ascending order from top to bottom.

We arbitrarily cropped it to train the data we have whenever an ancient document entered the network. In addition, we chose 1024 × 1024 as the closest power of two to 854 and 1253 pixels which are the average width and height of 40 ancient documents. At this point, we resize it while retaining the aspect ratio and then crop if the minimum width or height of ancient documents did not meet 1024. We evaluated the proposed method in the same manner as training, but we cropped sequentially from top left to bottom right to consider the entire page. At this step, we overlapped the cropped image by 512 pixels to compensate for the characters that were cut due to cropping. If the cropped image does not reach 1024 pixels in size, we maintain the aspect ratio by adding white padding to the lower right corner.

We pretrained the coordinate and score layers of the RPN Network at a learning rate of 10^−3^ for detector learning, and we trained the index layer (red box in Figure 3 green dash box) to minimize *L*_*in* *de* *x*_ (Equation (3) for ordering. We used Adam optimizer [46] with the learning rate of 10^−5^, *λ*_*line*_=10, *λ*_*y*_=1, batch size 1, and epoch number 15000 on Pytorch v1.8.0 with CUDA v11.3, CuDNN 8.0.2, and NVIDIA GTX 1080 TI.

### 4.2. Effectiveness of Two Dimensional Indices

We calculated the edit distance depending on the number of training dimensions using the detector and dataset described in sec. Experimental Setup to see how much more accurate the order value is assigned by the network learned with the line and *y*-axis separately versus the network learned with 1-dimensional indices. We evaluated character ordering without eliminating false detection which is a limitation of the detector. We may not remove the false alarm in actual cases because there may not be a correct label for the document data.

Our edit distance (ED) calculated the number of inaccurate indexings (Equation (6) in the original formula (equation (5) [43]) and the relative difference equation (7) between the order network predicted (pre d_*ind*_*i*_) and the ground truth order (target_*ind*_*i*_) for the i-th character. Furthermore, we give the wrong index number (*ED*′) a higher priority than the relative difference (*L*1) by multiplying it (*ED*′) by the number of letters (*N*) as indicated in equation (8). Then we divide that by the number of characters to get the ED values for each character. The lower the value, the greater the similarity to the correct answer because this value denotes string similarity.

(5) $$\begin{array}{c} ED^{\prime }=\overset{N}{\underset{i=1}{\sum }}\delta_{pred\_ind_{i},target\_ind_{i}}\text{,} \end{array}$$

(6) $$\begin{array}{c} \delta_{m,n}=\left\{\begin{array}{cc} 0\text{,} & ifm=n\text{,}\\ 1\text{,} & otherwise\text{,} \end{array}\right. \end{array}$$

(7) $$\begin{array}{c} L1=\overset{N}{\underset{i=1}{\sum }}pred\_ind_{i}-target\_ind_{i}\text{,} \end{array}$$

(8) $$\begin{array}{c} ED=\frac{N\times ED^{\prime }+L1}{N}\text{.} \end{array}$$

Table 1 shows the ED value for 100 test data sheets based on index dimensions. It shows the ED for the box corresponding to the correct character because there is no correct order corresponding to false detection. Table 1 (a) shows no character ordering, Table 1 (b) shows character ordering with 1-dim indices, and Table 1 (c) shows character ordering with 2-dim indices. If the output boxes of the detector are not rearranged, the order of the characters is determined at random (262.27). It decreases 0.77 times with 1-dim indices (203.49) and 0.42 times with 2-dim indices (108.86) when rearranged compared to the random arrangement.

This is because 1-dim indices have only one amount of information available to learn one index, but 2-dim indices (Figures 2(b) and 2(c)) have expanded the quantity of information by picking data for each line and *y*-axis of the ancient document (a combination of *S*_*h*_^*S*_*w*_^ and *S*_*w*_^*S*_*h*_^) as mentioned in sec. Two dimensional indices and network. The width and height of the feature map are represented by *S*_*w*_ and *S*_*h*_, respectively.

Another reason is that the learning range is restricted, and the learning complexity is reduced by dividing the learning dimension into *x*-axis and *y*-axis. We can know that the order is better assigned as the learning dimension increases, so the following experiment was conducted with 2-dimensional indices.

### 4.3. Effectiveness of Absolute Ground Truth

To determine which the ground truth (GT) map best optimizes the network for ordering, we compared the ED of the network trained using our absolute GT to the ED of the network trained using the original GT [40–42]. The ED of the network trained using the original GT is 108.86 (Table 1 (c)), and the ED of the network trained with our absolute GT is 0.25 (Index (c) in Table 2) indicating that our method had a lower ED. This is because our absolute GT facilitates network learning by labeling similar values at similar positions.

We analyzed the GT maps for two example papers with drastically varied text areas to see how different values are labeled at similar positions according to the GT generating approach. Figures 4(a) and 4(b) depict the GT lines given to each character in two different documents, one for the original GT and one for our absolute GT. We compared the value of the third line for each document. The line values of the two documents are 0.30 and 0.32 respectively with a difference of only 0.02 if we use our absolute GT, but the line values are 0.22 and 0.50 with a difference of 0.28 if we use the original GT. This indicates that similar values are labeled at similar positions throughout all papers when the absolute location is considered when constructing GT. But if the absolute position is ignored, the different values are tagged to characters even at similar locations between documents because consecutive ids are assigned. We employed our absolute GT to help to learn for our method because we found this tendency in other ancient documents.

### 4.4. Performance between Human-Designed Algorithm and Our Method

We compute the average edit distance (ED) and fisher criterion (FC) for 100 ancient documents to evaluate the ordering performance of our method. Afterward, we compare the performance of the human-designed algorithm and our method in Performance for Real Ancient Documents.

We prevented underfitting by increasing the number of epochs so that the model learned training data sufficiently, and we prevented overfitting by stopping training when the loss on validation data increased or converged during training. Furthermore, we demonstrated that underfitting and overfitting were avoided through the experiment result of a low edit distance and a high fisher criterion in Performance for Real Ancient Documents. However, even if the experimental results are good, overfitting still can be suspected when the train and test data of the ancient document are very similar. In this regard, we demonstrated high ordering performance for synthesized data with low similarity between the train and test data in sec. Performance for synthesized documents with split lines, proving that overfitting does not occur even when the train and test data are not similar.

### 4.5. Character Ordering Based Human-Designed Algorithm

The human-designed algorithm (Algorithm 1) demonstrates the step-by-step process of assigning indexes by allocating 2-dimensional indices using the coordinates and sizes of boxes placed in a random order such as sec. Two dimensional indices and network in our method. We define a collection to gather line values (*L*) and set the first box line id (*b*_1_) to 0 (line 8) to assign line ids based on the coordinates and sizes (*B*) of randomly arranged boxes. Then we measure the Euclidean distance [47] between the coordinates to be ordered and the coordinates already been ordered (line 14 and 15). We assign the same order (line 17) if the distance between the characters satisfies two conditions (Equations (9) and (10)) otherwise we assign a new order (line 20) and collect in the set *L*.

(9) $$\begin{array}{c} \;|\;x_{t}-x\;|\;\leq w_{t}\times threshold\text{,} \end{array}$$

(10) $$\begin{array}{c} \sqrt{{\left(x_{t}-x\right)}^{2}+{\left(y_{t}-y\right)}^{2}}<\;\min\text{.} \end{array}$$

Equations (9) and (10) indicate the conditions for measuring the closeness of two characters in order to assign the correct line ids. Given the coordinates of the box already ordered (*x*_*t*_, *y*_*t*_, *w*_*t*_, *h*_*t*_) and the coordinates of the box to be ordered (*x*, *y*, *w*, *h*), we calculate both the difference between the *x* coordinates of two boxes (Equation (9)) and the difference between the center coordinates (Equation (10) Then assign the same line ids if the values are less than the constraint condition. The threshold in equation (9) is a ratio that reduces *w*_*t*_ and conducts flexible line clustering by modifying the threshold based on the width of the box rather than limiting the fixed threshold based on the distance between the *x* coordinates. As indicated in line 18 of algorithm 1, the min in equation (10) is the least value of the distance between center coordinates and is updated whenever there is the nearest character. To make robust in the diagonal direction, these equations consider the center coordinates of all boxes and assign the same order as the nearest box previously had.

We sort the line ids assigned from the network to cluster if they are in the same order in the set *L*, and we sort again to maintain their correspondence with the set of detection boxes using the order (*in* *d*) used for sorting (line 21 and 22). The same id is assigned if it is in the same line, but the first line is assigned a random order not zero. So, we reassign a sequential order from right to left, identical to the translation order, for each line (line 23). Then we sort values from small to large for each line using *y*-axis ids (line 25), and we maintain their correspondence with the set of detection boxes (line 26). Finally, we assign consecutive values using ids separated by line and *y*-axis.

### 4.6. Performance for Real Ancient Documents

**Figure 6:**
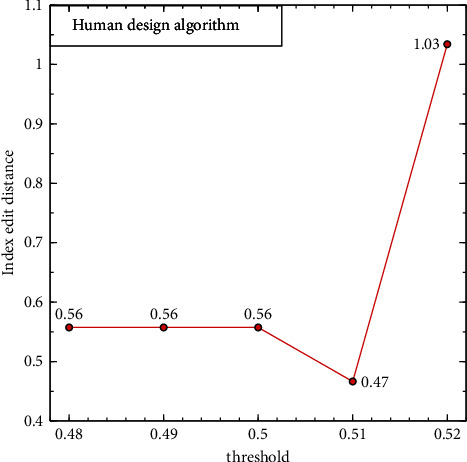
Index edit distance based on the human-designed algorithm.

**Figure 7:**
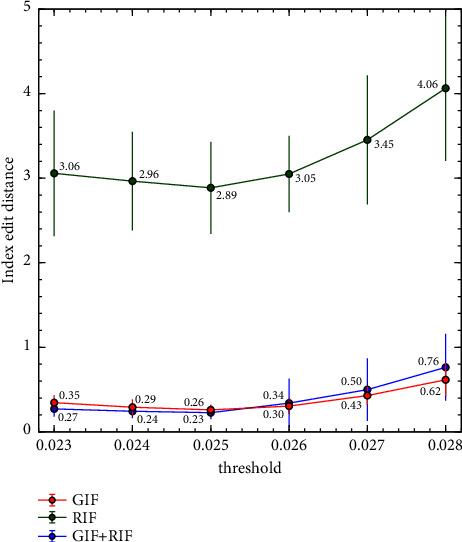
Index edit distance based on the input feature map of our method.

To confirm the trustworthiness of the learning-based model, we train ten networks for the input feature maps, i.e., graphical input feature map (GIF), ROI input feature map (RIF), and both feature map (GIF + RIF), specified by sec. ROI transfer while testing the performance of our method. Then we calculated the index ED for each input feature map by changing the threshold to find the appropriate threshold in equation (4) of our method and equation (9) of the human-designed algorithm. Figures 6 and 7 depict the average and standard deviation of the index ED based on the threshold. The human-designed approach performs best when the threshold = 0.51 regardless of the input feature map, while our method has the least index ED when the threshold = 0.025. So, we compare the performance using that threshold. Furthermore, the coefficients of variation (CV) of ten randomly initialized models in our method are relatively significant with 23, 19, and 33% in the order of GIF, RIF, and GIF + RIF. So, we compared the performance of the human-designed algorithm and our models except for one that had the greatest divergence from the average index ED for each method based on input feature map to minimize the CV to less than 20% so that the model could be assumed stable [48].

Table 2 compares the edit distances of the human-designed algorithm (Table 2 (a)) and our method (Table 2(b-d)) for 100 ancient documents with the threshold and number of models indicated in the preceding paragraph. Table 2 shows the type of edit distance in the first column, the method in the second column, the edit distance average (standard deviation) in the third column, and the ratio of our method to the human-designed algorithm in the fourth column (m/*a*, *m* = *b*, *c*, and *d*).

GIF (0.25) is lowered to 0.53 times that of the human-designed method (0.47) in Index ED (Index in Table 2). The reason is that it is necessary to find the rules of all patterns and enforce the conditions, but incorrect indexing may occur due to conditions not considered in advance when character ordering a handwritten document with a complex pattern such as a diagonal line or a line splitting into two lines with a human-designed algorithm. However, the learning-based network assigns more similar values in the same line than the human-designed algorithm, resulting in the correct line ids even with relatively few conditions because it infers a spatial relation.

The RIF index ED (2.79) is increased to 5.93 times that of the human-designed algorithm index ED (0.47). The reason for this is that RIF is a low-dimensional feature map extracted from GIF, and there is no information such as appearance, overlapping, or angle. Therefore, the right order cannot be determined.

However, the GIF + RIF index ED (0.20) which combines GIF and RIF is 0.43 times that of the human-designed algorithm index ED (0.47) and 0.8 times that of the GIF index ED (0.25). This is because GIF contains visual information that RIF does not have and RIF has the absolute position and a trend related to translation order. Thus, if coordinate values are similar, the network assigns similar ids corresponding to the translation order. We can see that our method outperforms the human-designed process in terms of ordering and that ordering improves as the amount of input feature information rises.

For line ED for each method, GIF (2.69) and RIF (11.92) are larger than the human-designed algorithm (2.28), and GIF + RIF (1.85) is smaller than the human-designed algorithm. The RIF (3.81) is larger than the human-designed algorithm (1.86) for the *y*-axis ED for each method, and GIF (1.64) and GIF + RIF (1.61) are smaller than the human-designed algorithm. In this case, the *y*-axis ED has the same trend as the index ED, but the line ED has a different tendency. Furthermore, the line ED value is very large while the index ED value for each method is small enough. Because an inaccurate line id is assigned to a character in the ancient document, all future orders are considered incorrect indexing regardless of the actual quantity of incorrect indexing.

As a result, we used the fisher criterion (FC) [44] to compare line ids performance with a more acceptable way for each method. It evaluates how close ids are if they are on the same line and how far apart ids are if they are on distinct lines on the document.

Original FC indicates how well it distinguishes between two classes in more than two dimensions space. In contrast, our FC indicates how well it distinguishes multiple classes in a one-dimensional space. This value is a ratio of the variance for each line, and the distance between *i*^th^ and *i*+1^th^ line averages among C lines and a larger value indicates better clustering.

(11) $$\begin{array}{c} S_{B}=\frac{1}{C-1}\cdot \overset{C-1}{\underset{i=0}{\sum }}{\left(\mu_{i}-\mu_{i+1}\right)}^{2}\text{,}\\ S_{W}=\overset{C}{\underset{i=1}{\sum }}\overset{1}{\underset{j=0}{\sum }}{\left(l_{j}-\mu_{i}\right)}^{2},\\ FC=\frac{S_{B}}{S_{W}}\text{.} \end{array}$$

We calculated FC in the same setting that we calculated ED. We used the normalized x coordinate of the detected box to [0, 1] for the human-designed algorithm data to evaluate performance in a fair scale setting, and we used line ids predicted by the network for our method data.

Table 3 shows the average FC (standard deviation) between the human-designed algorithm (Table 2 (a)) and our method (Table 2(b-d)). In this case, the GIF (47.36) is increased to 1.35 times that of the human-designed algorithm (35.10). RIF (24.76) has low FC due to lack of information as discussed in the ED results of Table 2. However, GIF + RIF (51.10) combined with GIF increases to 1.46 times. This shows that our method clusters line more easily than the human-designed algorithm and that line clustering improves as the amount of input feature information grows.

### 4.7. Performance for Synthesized Documents with Split Lines

We want to compare the performance of each method on a document with split lines where our method outperforms the human-designed algorithm. To do this, we created a document with split lines and estimated the index edit distance related to the final ordering performance such as Performance for Real Ancient Documents. In addition, we calculated a fisher criterion that measures the degree of line clustering which is a feature that impacts performance.

We used 100 character-free background images and individual CASIA [49] character images to create the synthesized data. When synthesizing a document, considerations include the size of the background image, the number and size of characters to be written in the background image, the number of lines that will split into two lines, and the distance between the lines divided into two lines.

We resized the shorter length of each background image at random between 1000 and 1400 pixels while maintaining the aspect ratio. In addition, we chose one of 0, 90, 180, or 240 degrees and rotated it to the augment data. We generated various sized characters by randomly selecting several characters ranging from 30 to 250 and randomly scaling each character to have a short length between 50 and 70 pixels.

We selected several lines divided into two lines that range from one to four per document. And the distance between the lines that split into two was calculated to be 0.3, 0.4, and 0.5 times the width of the characters in the line before the split. We generated 13 pieces of training data, 3 pieces of validation data, and 33 pieces of test data for each of these three ratios.

The first column in Table 4 is the index edit distance and fisher criterion, the second column is the method, the third column is the average of performance (standard deviation), and the fourth column is the ratio of our method to the human-designed algorithm (m/*a*, *m* = *b*, *c*, and *d*). Regarding Index ED, GIF (15.77), RIF (28.27), and GIF + RIF (14.86) are reduced to 0.52 times, 0.93 times, and 0.49 times, respectively, than the human-designed algorithm (30.42). Regarding FC, GIF (26.99), RIF (10.52), and GIF + RIF (40.29) are increased to 3.49 times, 1.36 times, and 5.21 times, respectively, than the human-designed algorithm (7.74). This result is similar to the index ED trend (human = 14.88, RIF = 9.79, GIF = 6.40, and GIF + RIF = 6.73) of documents that have split lines into two on ancient documents, and it is also similar to the FC trend (human = 4.00, RIF = 4.96, GIF = 13.46, and GIF + RIF = 18.73).

Some documents cannot be processed because the distance between the two lines is too close, resulting in a high index ED and standard deviation on our method. However, our method outperforms the human-designed algorithm regarding ordering performance. Furthermore, adding the absolute position and the trend corresponding to the translation order in the RIF to the graphic information of GIF in our method improves performance due to the effect of increasing the amount of input feature information.

### 4.8. Result Image

We compared several images of the human-designed algorithm and our method qualitatively. We used the threshold (0.51 for human-designed algorithm and 0.025 for our method) determined in Performance for Real Ancient Documents. To compare with the human-designed algorithm, we chose the model with the smallest deviation from the average index ED as shown in Table 2 from among the nine GIF, RIF, and GIF RIF models determined in sec. Performance for real ancient documents.

**Figure 8:**
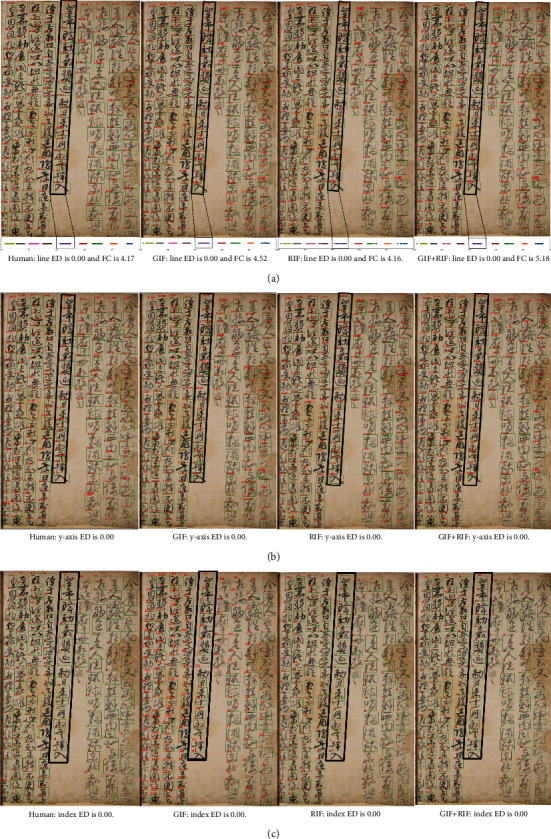
The ids assigned to an ancient document with diagonal direction. (a) The line ids. (b) The y-axis ids. (c) The index ids.

We compared the line ids (Figure 8(a)) of the human-designed algorithm (Human) and our method (GIF, RIF, and GIF + RIF) for an ancient document with diagonal lines. The upper part of these images shows the detected character box and line ids in the ancient document, and the lower part shows the line id dots are drawn using different colors for each line on the *x*-axis.

Line ED in the sample images is all 0.00 regardless of method and the characters corresponding to the diagonal lines (black boxes) are marked with the same color dots (purple) on the *x*-axis. We can find that documents with diagonal direction lines do well in line clustering regardless of the method.

Figure 8(b) is the results of assigning the *y*-axis ids to the ancient document and figure 8(c) is the results of giving a series of final index ids. We can find that both the human-designed algorithm (Human) and our method (GIF, RIF, GIF + RIF) assign the proper ids to the characters in the diagonal direction because two types of ED are 0.00 regardless of the method.

Regarding FC, GIF + RIF (5.18) and GIF (4.52) have higher values than the human-designed algorithm (4.17), and RIF (4.16) has the smallest value. As mentioned in sec. Performance for real ancient documents, our method has a larger FC as the amount of the input feature information increases and our method performs line clustering better than the human-designed algorithm in the case of GIF + RIF and GIF.

**Figure 9:**
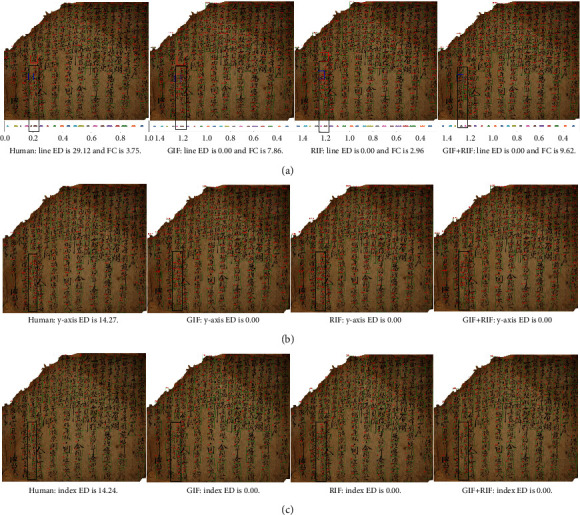
The ids assigned to an ancient document with a dividing line into two lines. (a) The line ids. (b) The y-axis ids. (c) The index ids.

For an ancient document with lines that split into two lines, we compared the line ids (Figure 9(a)) of the human-designed algorithm (Human) and the proposed method (GIF, RIF, and GIF + RIF). The upper and lower images are identical to those in Figure 8(a). Our method (GIF, RIF, and GIF + RIF) assigns the correct line ids to the characters written in two lines (black box) rather than the human-designed algorithm and displays them on the *x*-axis line as dots in different colors (pink and brown). On the other hand, the human-designed algorithm (Human) assigns same ids to characters written in two lines showing dots of the same color (brown) on the *x*-axis line. In addition, our method (GIF, RIF, GIF + RIF) assigns values that are larger than the threshold (0.025) determined in sec. Performance for real ancient documents to the line ids of characters as you can see from the red and blue boxes in each figure, whereas the human-designed algorithm (Human) assigns values that are smaller than the threshold (0.51). The line ED of our method (GIF, RIF, GIF + RIF) is 0.00, but the line ED of the human-designed algorithm (Human) is high as 29.12 because of this difference.

Figure 9(b) shows the *y*-axis id and Figure 9(c) shows the final index id. Our method indicates that it assigns rising numbers to the *y*-axis id from top to bottom for all lines, including the split line (black box). It also indicates that the correct index id is assigned to the line that has been split into two lines (black box). This shows that our method (GIF, RIF, and GIF + RIF) has lower *y*-axis ED and index ED and better ordering than the human-designed algorithm (Human).

GIF + RIF (9.62) and GIF (7.86) have greater FCs than the human-designed algorithm (3.75) and RIF (2.96) has the lowest FC. As stated in sec. Performance for real ancient documents, our method (GIF, RIF, and GIF + RIF) has a higher FC as the amount of input feature information rises, and GIF + RIF and GIF perform line clustering better than the human-designed algorithm (Human).

### 4.9. Processing Time

We tested the processing time for each method with the same environment as sec. Performance for real ancient documents to show whether real-time ordering is possible. However, we do not consider evaluating ordering performance in real case, because ordering performance can be influenced by factors other than the ordering module.  Table 5 shows the time consumption for the ordering pipeline. The first column of Table 5 shows the method, from the second to fifth columns show the time taken for network forwarding, line clustering, and the line and *y*-axis sorting respectively, and the sixth column shows the total time consumed.

There is no network forwarding process in the human-designed algorithm because it is not based on learning, whereas our method is based on learning, so there is a period for network forwarding. However, this time consumption is so small that it is not a problem in real-time processing. The process after network forwarding is a common process regardless of the method. There is a slight time difference because line clustering runs linearly in the human-designed algorithm and parallelly in our method. Apart from this procedure, our method takes less time demonstrating that it is capable of real-time processing.

## 5. Conclusion

In this paper, we proposed a learning-based character ordering to assign the correct orders on the characters detected in a random order for correct translation. Our method increased the amount of learning information with 2-dimensional indices and learned to assign similar ids with absolute ground truth which considers absolute position if the characters in the document are in similar positions. We also assigned line ids which can be clustered well using both graphical and ROI information as an input. We addressed a previously undiscovered problem, ordering character, suggested a solution, and performed ordering character in a document using a learning-based model that learns the necessary computing operations from the document data. Our method using the graphical input feature map reduced the index edit distance by 0.53 times and enhanced the fisher criterion by 1.35 times when compared to the human-designed algorithm for 100 actual ancient documents. Furthermore, adding ROI information to our method reduced the index edit distance by 0.43 times and increased the fisher criterion by 1.46 times compared to the human-designed algorithm.

We first suggested an approach to the character ordering problem, and we outperformed the human-designed algorithm in character ordering. In particular, ours ordering outperformed the human-designed algorithm for documents with split lines into two. For the horizontally written documents, our method works for them by applying once we have horizontal character orders as ground truth and make absolute ground truth in horizontal direction.

However, we have some misordering of documents with split lines and overlapping boxes. Misordering of some overlapping bounding boxes is caused by the incorrect result of box detection network. Once overlapping boxes are detected, the incorrect boxes may cause temporary incorrect orders. However, the relative orders of the following characters are not affected. For the misordering on the split lines, dealing with this problem will be our future work.

## Figures and Tables

**Algorithm 1:**
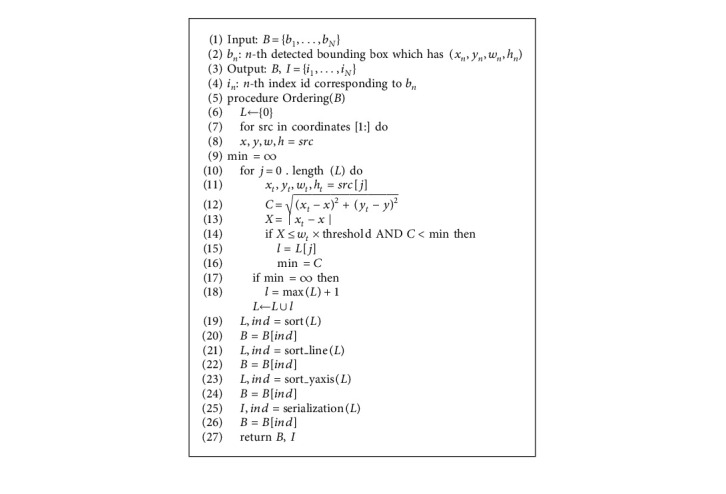
Human-designed algorithm.

**Table 1:** ED represents the edit distance between 1-dimensional and 2-dimensional index. The difference (m/*a*, *m* = *a* or *b* or c) represents the ratio between methods.

	Dimension of index	ED	Difference
W/*o* character ordering	(a) 1-dimensional indices	262.27	1

w character ordering	(b) 1-dimensional indices	203.49	0.77
	(c) 2-dimensional indices	108.86	0.42

**Table 2:** Average edit distance (standard deviation) of the human-designed algorithm (a) and our method (b, c, and d). Difference rate indicates the ratio between methods (m/*a*, *m* = *b*,c, and d).

Type of indexing	Method	Edit distance ↓	Difference rate
Line	(a) Human-designed	2.28	1
	(b) RIF	11.92 (3.20)	5.23
	(c) GIF	2.69 (1.03)	1.18
	(d) GIF + RIF	1.85 (0.17)	0.81

*Y*-axis	(a) Human-designed	1.86	1
	(b) RIF	3.81 (0.51)	2.05
	(c) GIF	1.64 (0.06)	0.88
	(d) GIF + RIF	1.61 (0.05)	0.87

Index	(a) Human-designed	0.47	1
	(b) RIF	2.79 (0.49)	5.93
	(c) GIF	0.25 (0.05)	0.53
	(d) GIF + RIF	0.20 (0.01)	0.43

**Table 3:** Average fisher criterion (standard deviation) of the human-designed algorithm (a) and our method (b, c, and d). Difference rate indicates the ratio between methods (m/*a*, *m* = *b*,c, and d).

Method	Fisher criterion ↑	Difference rate
(a) Human-designed	35.10	1
(b) RIF	24.76 (1.59)	0.71
(c) GIF	47.36 (2.72)	1.35
(d) GIF + RIF	51.10 (4.20)	1.46

**Table 4:** Average index edit distance or fisher criterion (standard deviation) of the human-designed algorithm (a) and our method (b, *c*, and d). Difference rate indicates the ratio between methods (m/*a*, *m* = *b*,c, and d).

Type of performance	Method	Performance	Difference rate
Index edit distance ↓	(a) Human-designed	30.42 (17.61)	1
	(b) RIF	28.27 (17.95)	0.93
	(c) GIF	15.77 (15.11)	0.52
	(d) GIF + RIF	14.86 (15.41)	0.49

Fisher criterion ↑	(a) Human-designed	7.74 (5.92)	1
	(b) RIF	10.52 (7.29)	1.36
	(c) GIF	26.99 (39.60)	3.49
	(d) GIF + RIF	40.29 (60.74)	5.21

**Table 5:** Average (Standard deviation) running times (sec) for each module in the ordering pipeline: (a) The Human-designed algorithm, (b) RIF, (c) GIF, (d) GIF + RIF.

	Network forwarding	Line clustering	Line sorting	Sorting *y*-axis	Total
(a) Human-designed	N/A	4.3471 (4.4089)	0.0016 (0.0018	0.0001 (0.0000)	4.3487 (4.4107)
(b) RIF	0.0262 (0.0107)	0.0029 (0.0008)	0.0003 (0.0001)	0.0001 (0.0000)	0.0294 (0.0116)
(c) GIF	0.0289 (0.0111)	0.0031 (0.0009)	0.0003 (0.0001)	0.0001 (0.0000)	0.0324 (0.0120)
(d) GIF + RIF	0.0295 (0.0112)	0.0032 (0.0010)	0.0003 (0.0001)	0.0001 (0.0002)	0.0331 (0.0125)

## Data Availability

The data used to support the findings of this study are available from the corresponding author upon request.
